# Imaging-derived biomarkers from ^68^Ga-DOTATOC PET/CT scans to predict survival of patients with neuroendocrine tumors after PRRT with ^177^Lu-DOTATATE

**DOI:** 10.1186/s40644-025-00899-5

**Published:** 2025-07-01

**Authors:** Stephan Raad, Ali Al-Fatlawi, C. Louise Wise, Christian Fottner, Simin Schadmand-Fischer, Mathias Schreckenberger, Matthias M. Weber, Thomas J. Musholt, Michael Schroeder, Matthias Miederer

**Affiliations:** 1 National Center for Tumor Diseases (NCT), NCT/UCC Dresden, A Partnership Between DKFZ, Faculty of Medicine and University Hospital Carl Gustav Carus, TUD Dresden University of Technology, and Helmholtz- Zentrum Dresden-Rossendorf (HZDR), Dresden, Germany; 2 Biotechnology Center (BIOTEC), Center for Molecular and Cellular Bioengineering, Technische Universität Dresden, Dresden, Germany; 3 Department of Nuclear Medicine, University Medical Center of the Johannes Gutenberg University, 55131 Mainz, Germany; 4 Department of Endocrinology, University Medical Center of the Johannes Gutenberg University, 55131 Mainz, Germany; 5 Department of Radiology, University Medical Center of the Johannes Gutenberg University, 55131 Mainz, Germany; 6 Department of Surgery, University Medical Center of the Johannes Gutenberg University, 55131 Mainz, Germany; 7 University of Kufa, Kufa, Iraq; 8 Center for Scalable Data Analytics and Artificial Intelligence (ScaDS. AI), Dresden, Germany

**Keywords:** Neuroendocrine tumors, PRRT, ^68^Ga-DOTATOC PET/CT, Tumor volume, SUV, Radiomics, Overall survival

## Abstract

**Background:**

Neuroendocrine tumors have increased in prevalence and diversity in recent years and are often diagnosed at metastatic stages. Compared with nonradioactive systemic treatment with somatostatin analogs, peptide receptor radionuclide therapy (PRRT) has shown superior overall survival benefits for well-differentiated neuroendocrine tumor patients. This study aimed to identify biomarkers from ^68^Ga‒DOTATOC PET/CT scans to predict survival in patients treated with PRRT in the clinic.

**Methodology:**

This retrospective study analyzed ^68^Ga-DOTATOC PET/CT data from 67 NET patients undergoing PRRT. Tumor volumes and SUV metrics were segmented using standardized protocols. Radiomics features from liver metastases were extracted and preprocessed for analysis. Data were analysed via Kaplan-Meier, Cox regression, and PCA to evaluate the prognostic value of volumetric-, radiomics-, and clinicopathological parameters.

**Results:**

This study included scans from 67 patients with an average age of 67 years. The mean survival time was 46.5 months, with 43% of patients alive or lost to follow-up at the conclusion of data collection. Despite comprehensive analyses, neither volumetric parameters, including total tumor volume and organ-specific tumor volume, nor SUV values (SUVmax and SUVmean) were robust predictors of overall survival. K‒M and Cox regression analyses revealed no significant differences in survival between the high- and low-risk groups for these parameters. Furthermore, radiomics features extracted from liver metastases did not demonstrate significant prognostic value.

**Conclusion:**

Quantification of ^68^Ga-DOTATOC PET/CT-derived parameters offers limited prognostic value for OS in NET patients who are receiving PRRT in clinical practice. These findings might emphasize the current robust integration of imaging in clinical decision-making for NET management.

## Introduction

Neuroendocrine tumors (NETs) have become more prevalent in recent years, despite being historically considered rare [1, 2]. These tumors can originate from various organs and differ significantly in their biological behavior and degree of differentiation [3]. NETs are categorized into three grades: well-differentiated low-grade (G1), well-differentiated intermediate-grade (G2), and poorly differentiated high-grade (G3) [3].

High somatostatin receptor (SSR) expression, characteristic of most well-differentiatied NETs, has enabled major advances in imaging and therapy [1]. Most patients present with metastatic disease at diagnosis, making treatment selection a key determinant of overall survival (OS) [4]. Among systemic therapies, peptide receptor radionuclide therapy (PRRT) has shown superior outcomes compared to alternatives [5, 6].

With growing therapeutic options, it is crucial to identify prognostic and predictive markers for personalized treatment planning. Clinical biomarkers, such as chromogranin [7], the inflammation-based index (IBI) [8], alkaline phosphatase (ALP), and lactate dehydrogenase (LDH) [9] have been proposed. At the same time, ^68^Ga-DOTATOC has become essential in NET management due to its high sensitivity and ability to quantify tumor burden and SSR expression [10, 11]. 

Furthermore, radiomics, the extraction of high-dimensional features from imaging data, offers a noninvasive method to evaluate tumor heterogeneity. Applied to SSR-PET, radiomic features may outperform conventional metrics such as SUVmax or SUVmean in prognostic accuracy [12, 13]. Additionally, these features possess the ability to differentiate between high-risk and low-risk groups designated for PRRT, thereby offering a refined approach to patient stratification and management [13].

This study aimed to evaluate the prognostic value of quantitative and radiomic parameters derived from DOTATOC PET/CT in patients with NET undergoing PRRT. Specifically, we assessed: First, the association between total and organ-specific tumor volume and overall patient survival following PRRT. Second, the prognostic value of SUVmax and SUVmean, both for the total tumor load or organ-specific, in relation with OS. Third, the prognostic ability of radiomic features applied to liver metastases in predicting patient outcomes. Finally, we aimed to determine whether clinicopathological parameters such as sex, age, and the number of organs affected may predict OS.

## Methodology

### Study population

In this study, we retrospectively analyzed data from 70 patients diagnosed with NETs who underwent DOTATOC PET/CT imaging from 2009 to 2016 at the University Hospital of Mainz, Germany, before receiving PRRT with Lutetium-177-DOTATATE. The median time from DOTATOC PET to treatment initiation was 1.01 months (IQR: 1.77). This study followed informed consent protocols and received approval from the local ethical committee. We excluded 3 patients due to a very short period of follow-up (< 6 months). Overall survival (OS) was defined as the time interval from the date of the first ^177^Lu-DOTATATE therapy session to the date of death or last follow-up for surviving patients.

### Imaging protocols

The imaging protocol for ⁶⁸Ga-DOTATOC PET/CT includes premedication with 20 mg of furosemide and 500 mL of Ringer’s solution (to minimize radiation dose), adjusted according to the patient’s preexisting conditions. Before the procedure, patients are instructed to empty their bladder and reduce physiologic urinary tracer activity, although fasting is not required.

During the scan, patients are positioned supine with their arms placed above their head. Image acquisition is performed 45 to 90 min after the injection of the radiotracer, with the scan covering the area from the vertex to the mid-thigh. In older protocols, the scan begins at the lower orbit (skull base).

The radiotracer used is ⁶⁸Ga-DOTATOC, administered at a dosage of 2.0 MBq/kg. The procedure utilizes the GEMINI TF by Philips, a hybrid scanner combining multislice computed tomography (16 lines) with positron emission tomography (time-of-flight PET). The scanner operates with standard voltage settings of 90 kV and 60 mAs.

Imaged were reconstructed using the BLOB-OS-F reconstruction method with a matrix size of 144 × 144 and a slice thickness of 4 mm. For analysis low dose CT attenuation corrected images were used.

### Segmentation

Using Snygo via VB80 software and the MI General workflow, we segmented the lesions in each PET/CT image for each patient. We began by manually placing a 3 cm spherical VOI in a healthy liver segment, enabling the lesion scout tool to perform automatic lesion segmentation. Eight patients had extensive liver metastases, preventing us from drawing the VOI in the liver. Therefore, we used the aorta as the reference organ.

For tumor volume measurement, we selected the lesions with an SUVpeak higher than the PERCIST-Liver value (1.5*mean + 2 standard deviations from the VOI drawn in the liver) [12]. Liver background as threshold was used to ensure high probability for focal uptake corresponding to metastasis and therefore decreasing the number of manual correctione steps. We then applied a segmentation threshold of 42% local SUVmax [14]. We excluded all lesions smaller than 0.5 mL. Physiological false positives were manually removed, and lesions not automatically selected were semi-automatically delineated with the VOI Isocontour tool using the same segmentation threshold.

We then used the Organ Segmentator tool to categorize lesions into liver (LV), bone (BV), lymph node (LNV) and abdominal (AV) regions, including mesenteric and peritoneal metastases. Lesions anterior to the aorta were classified as abdominal lesions, whereas those posterior or lateral to the aorta were classified as lymph nodes. Additionally, we measured the liver disease burden (LDB), which is the relative tumor as a percentage of the liver tumor volume in the liver organ volume.

### SUV

The SUVmean and SUVmax values were automatically generated for each lesion from Syngo.via VB80. The overall SUVmean of the patient was calculated in Microsoft Excel (Version 2016) for total tumor mass as well as the individual organ systems. This was achieved by averaging all the SUV means of each lesion relative to the corresponding tumor volume.

### Radiomics

For texture analysis, we segmented liver metastases via the PET segmentator tool in 3D Slicer 5.6.2. Before feature extraction, images underwent preprocessing steps to standardize the data. These steps included interpolation to obtain uniform voxel sizes and binning to discretize intensity values for consistent radiomics calculations. We utilized radiomic features of the three largest liver metastases for each patient, focusing on patients with more than 10 liver metastases, resulting in the inclusion of 46 patients in this analysis. For each patient, we computed a volume-weighted average of the radiomic features from the three largest lesions to derive a representative radiomic profile. This method was selected based on evidence which demonstrated that volume-weighted aggregation of radiomic features provides a more accurate and reliable representation of tumor heterogeneity in cases with multiple lesions, particularly when the number of lesions exceeds 10. The volume-weighted approach accounts for the disproportionate influence of larger lesions on overall tumor burden and radiomic signatures, ensuring that the derived profile reflects the dominant characteristics of the tumor population while minimizing bias introduced by smaller lesions [15].

The radiomic parameters that were extracted were divided into eight groups according to the definition of PyRadiomics: first-order statistics, shape-based (3D), shape-based (2D), gray level co-occurrence matrix, gray level run length matrix, gray level size zone matrix, neighboring gray-tone difference matrix, and gray level dependence matrix.

### Statistical analysis

We evaluated the associations between radiomic-, volumetric-, uptake-, clinicopathological features and overall survival via statistical tests.

The Kaplan‒Meier survival probabilities were calculated with Kaplan–MeierFitter (Lifelines library, Python 3.10.12), and survival differences between the high- and low-risk groups were analyzed via the log-rank test, with p values reported with SPSS v29.0. ROC curve analyses (Scikit-learn, Python 3) were performed to identify optimal thresholds for risk stratification.

We conducted univariate Cox regression analysis with SPSS v29.0. This approach is critical for identifying potential prognostic factors that could influence patient survival outcomes.

Given the small group size of the cohort, kernel density estimation (KDE) plots (seaborn library in Python 3.10.12) were generated to visualize the distribution of volumetric, uptake and radiomic features across survival groups (≤ 36 months, 36–60 months, and > 60 months). Patients with missing or zero values were excluded from each feature analysis.

For multivariable analysis, principal component analysis (PCA) was applied to radiomic features to reveal underlying patterns. Binary survival classes were created for the 36- and 60-month thresholds, with ROC curves and area under the curve (AUC) values calculated for each principal component to evaluate survival group discrimination.

## Results

### Sociodemographic

**Fig. 1:**
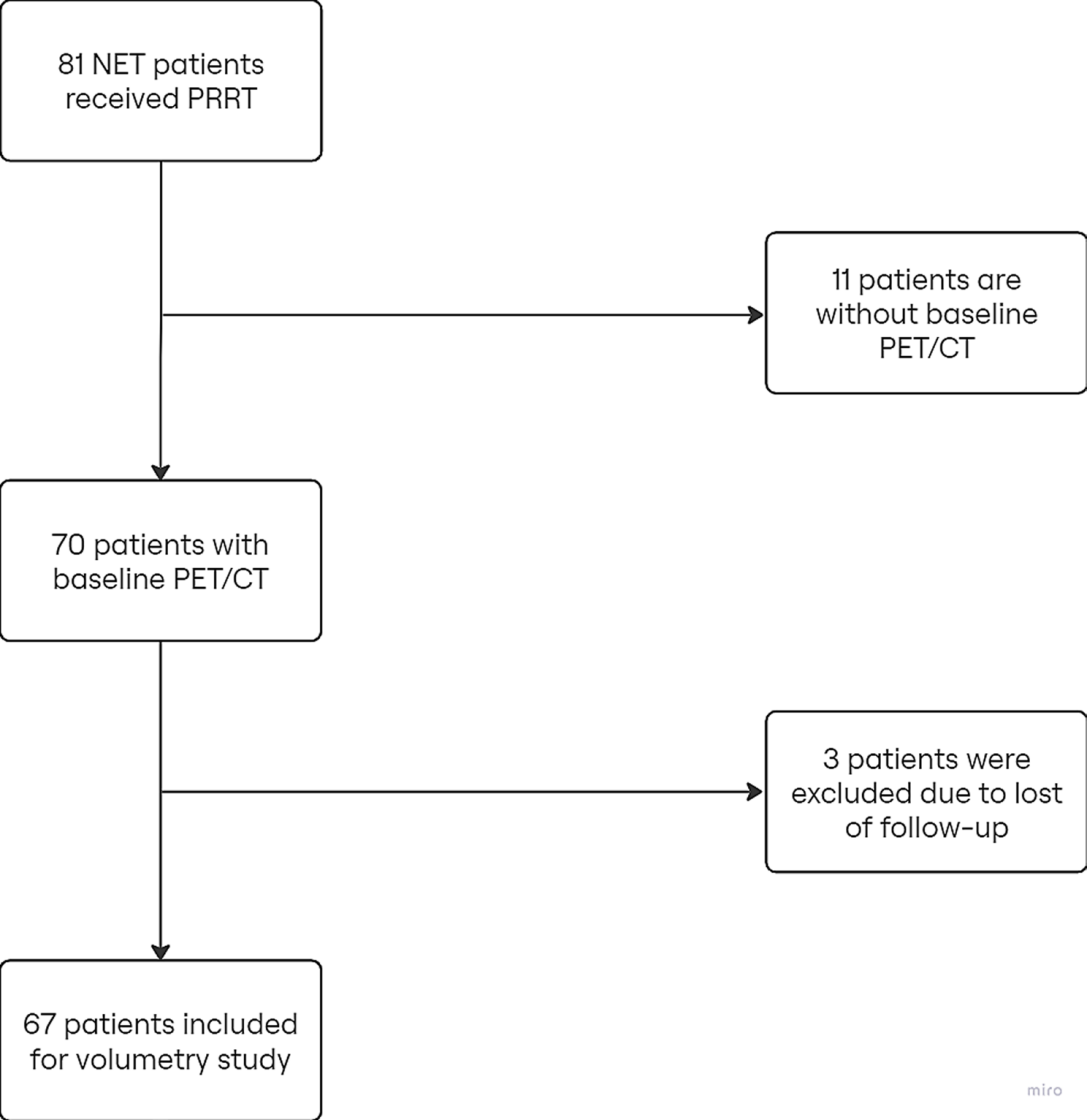
The course of the study

The study included 67 patients (Fig. 1) with a mean age of approximately 66.8 years. The sample consisted of 39 males (58.2%) and 28 females (41.8%). In terms of survival status, 29 patients (43.3%) survived, while 38 patients (56.7%) died. The average survival time across the cohort was 46.46 months with a median of 47.5 months. Most patients received ^177^Lu-DOTATATE PRRT (59/67), with only 8/67 patients receiving a combination of ^177^Lu-DOTATATE and ^90^Y-DOTATOC. With respect to grading, 23.9% were graded as 1, 46.6% as 2, and 29.9% had missing grading information. The primary tumor sites were predominantly in the small intestine (41.8%) and pancreas (29.9%), with fewer cases in the lung (7.5%) or other locations (including stomach, rectum, thymus, breast, kidneys and the liver) (20.9%). The spread of metastases varied, with the majority involving two (38.8%) or three organ systems (32.8%) (Table 1).

For each parameter in the survival analysis, we divided the patients into high and low groups using the median value as the cutoff.

**Table 1:** Sociodemographic characteristics, survival time, grade, primary tumor site and spread of metastases

Parameter	Categories	*N*	Percent
Age Median (IQR)		66,83 (59.75–75.25)	
Sex	Male	39	58,2
	Female	28	41,8
Survival Status	Alive	29	43,3
	Dead	38	56,7
Survival Time (months)	Alive	62.7	
	Dead	42.5	
	Overall	54.1	
Type of PRRT	Lu177	59 (3.36 cycles on average)	88,1
	Lu177 + Y90	8	11,9
Grading	1	16	23,9
	2	31	46,6
	Missing	20	29,9
Primary Tumor Site	Small intestine	28	41,8
	Pancreas	20	29,9
	Lung	5	7,5
	Other	14	20,9
Spread of Metastases	1 Organ system	5	7,5
	2 Organ systems	26	38,8
	3 Organ systems	22	32,8
	4 Organ systems	14	20,9

### Volumetric frequencies

Among the 67 patients, 61 had liver metastases, 36 had lymph node metastases, 48 had abdominal metastases, 37 had bone metastases, and 5 had metastases in other organs. The mean total volume of metastases was 271.03 mL. Specifically, the mean liver volume was 223.19 mL, the mean lymph node volume was 21.8 mL, the mean abdominal volume was 25.9 mL, and the mean bone volume was 65.61 mL. Other parameters including mean LDB was 9.84% (Table 2).

**Table 2:** Volumetric parameters with the number of patients analyzed and the mean values for each parameter

Parameter	Number of Patients	Mean Volumes ml	Volume Range {ml} (min - max)
Total Tumor Volume (TV)	67	271.03	1232.19 (1.89–1233.98)
Liver Tumor Volume (LV)	61	223.19	1162.391 (1.09–1164)
Bones Tumor Volume (BV)	37	65.61	919.49 (0.51–920)
Lymph Nodes Tumor Volume (LNV)	36	21.8	116.42 (0.96–117.38)
Abdominal Tumor Volume (AV)	48	25.9	89.15 (1.02–90.17)
Others	5	1.52	69.11 (1.35–70.46)

### Survival analysis

Univariate Cox regression analysis indicated that none of the volumetric parameters- including TV, LV, AV, BV, LNV, and LDB- were significantly associated with OS (all *p* > 0.05) (results detailed in Table 3).

**Fig. 2:**
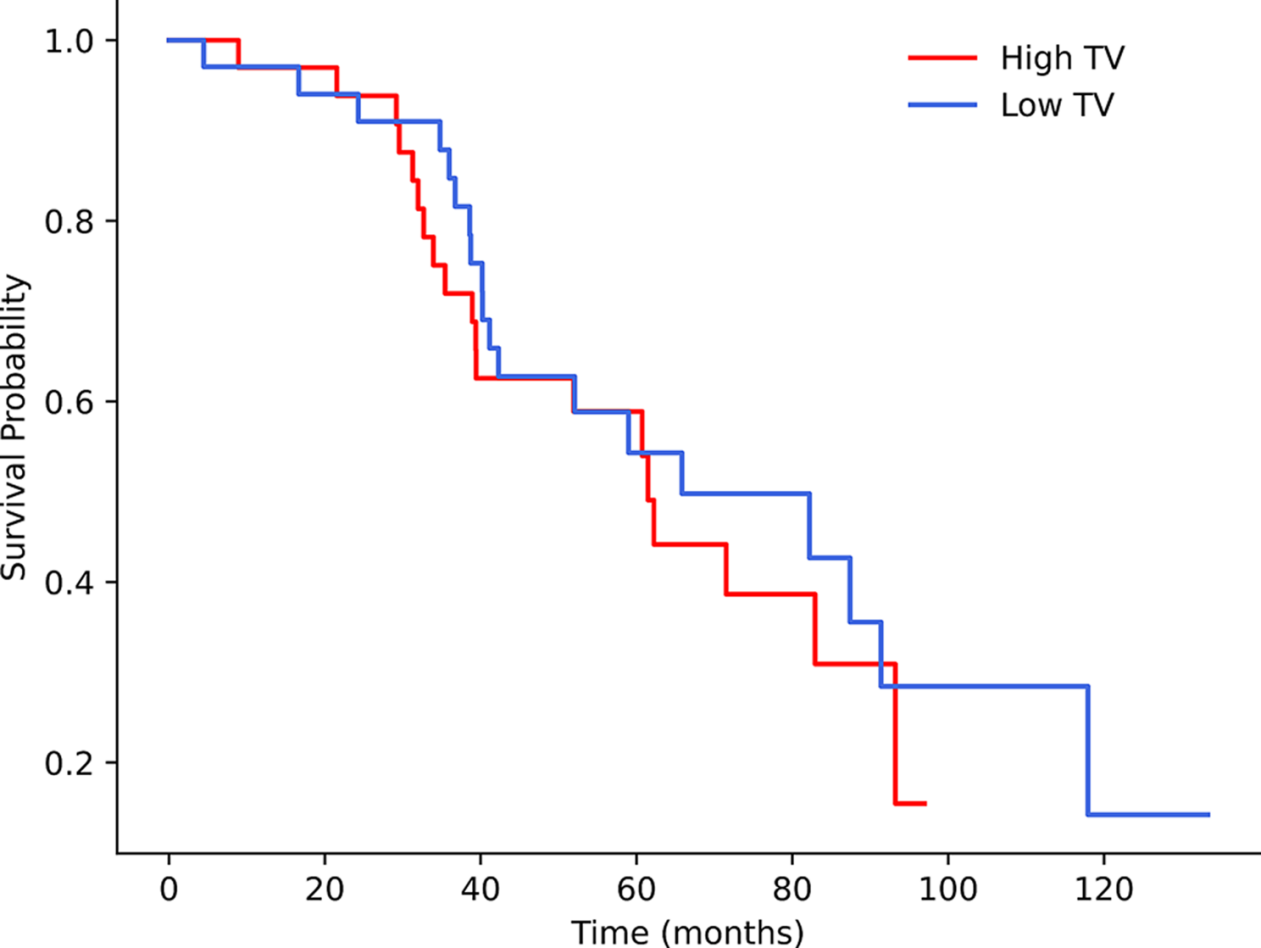
Kaplan‒Meier survival analysis showing high TV and low TV curves

**Fig. 3:**
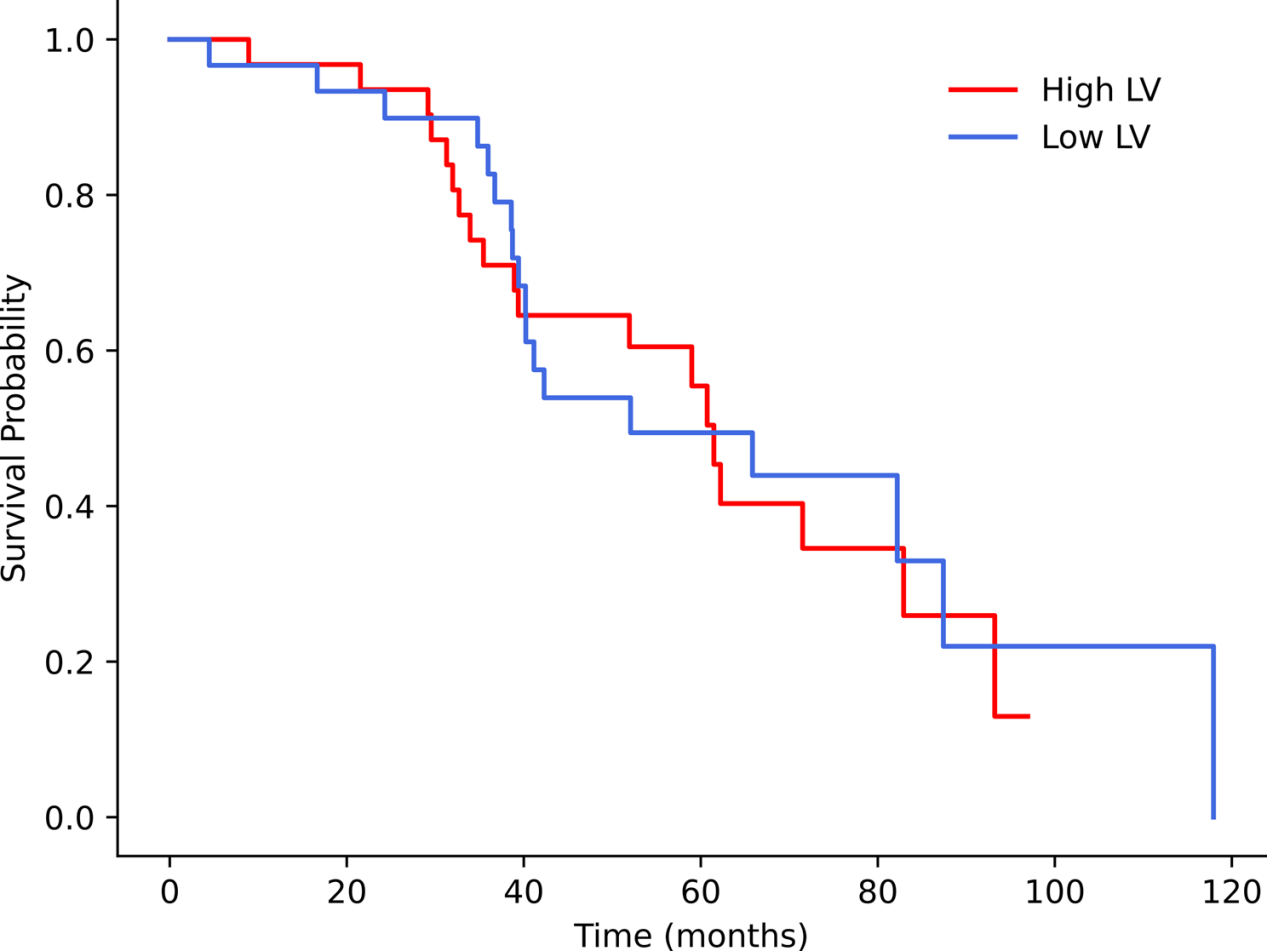
Kaplan‒Meier survival analysis showing high-LV and low-LV curves

Kaplan-Meier analysis showed no significant differences between these two groups across all volumetric parameters. However, patients in high-volume groups generally had shorter mean survival times, though this trend did not reach significance (Table 4) (Figs. 2 and 3).

**Table 3:** Cox regression analysis for volumetric parameters

Parameter	*p* Value	HR (95% CI)
TV	0.541	1.000 (0.999–1.001)
LV	0.815	1.000 (0.999–1.001)
AV	0.533	1.005 (0.989–1.022)
BV	0.752	0.999 (0.995–1.003)
LNV	0.754	1.002 (0.988–1.017)
LDB	0.382	1.013 (0.984–1.044)

**Table 4:** Survival analysis for volumetric parameters

Parameter	Median	Mean Survival (95% CI) low group vs. high group	Survival Rate (low group vs. high group)	Log-rank – Mantel Cox (*p* value)	Number of Patients
TV	150 mL	74.29 (59.2-89.39) mo **vs. 62.34 (52.21–72.46)** mo	44% **vs. 42.4%**	0.538	67
LV	120 mL	65.99(51.1–80.8) mo **vs. 61.02 (50.97–71.08)** mo	40% **vs. 38.7**%	0.898	61
BV	11.5 mL	80.54(61.5-99.54) mo **vs. 61.81 (52.39–71.23) mo**	42.1% **vs. 50%**	0.514	37
AV	21.4 mL	73.75 (58.79–88.6) mo **vs. 58.41 (45.98–70.84)** mo	49% **vs. 29.4%**	0.363	48
LNV	9.02 mL	73.64 (56.57–90.7) mo **vs. 61.13 (50.5–71.7)** mo	46% **vs. 37.5%**	0.865	36
LDB	6%	71.11 (54.55–87.67) mo **vs. 57.45 (48.4–66.4)** mo	44.4% **vs. 25%**	0.306	61

### KDE analysis for volumetric features

**Fig. 4:**
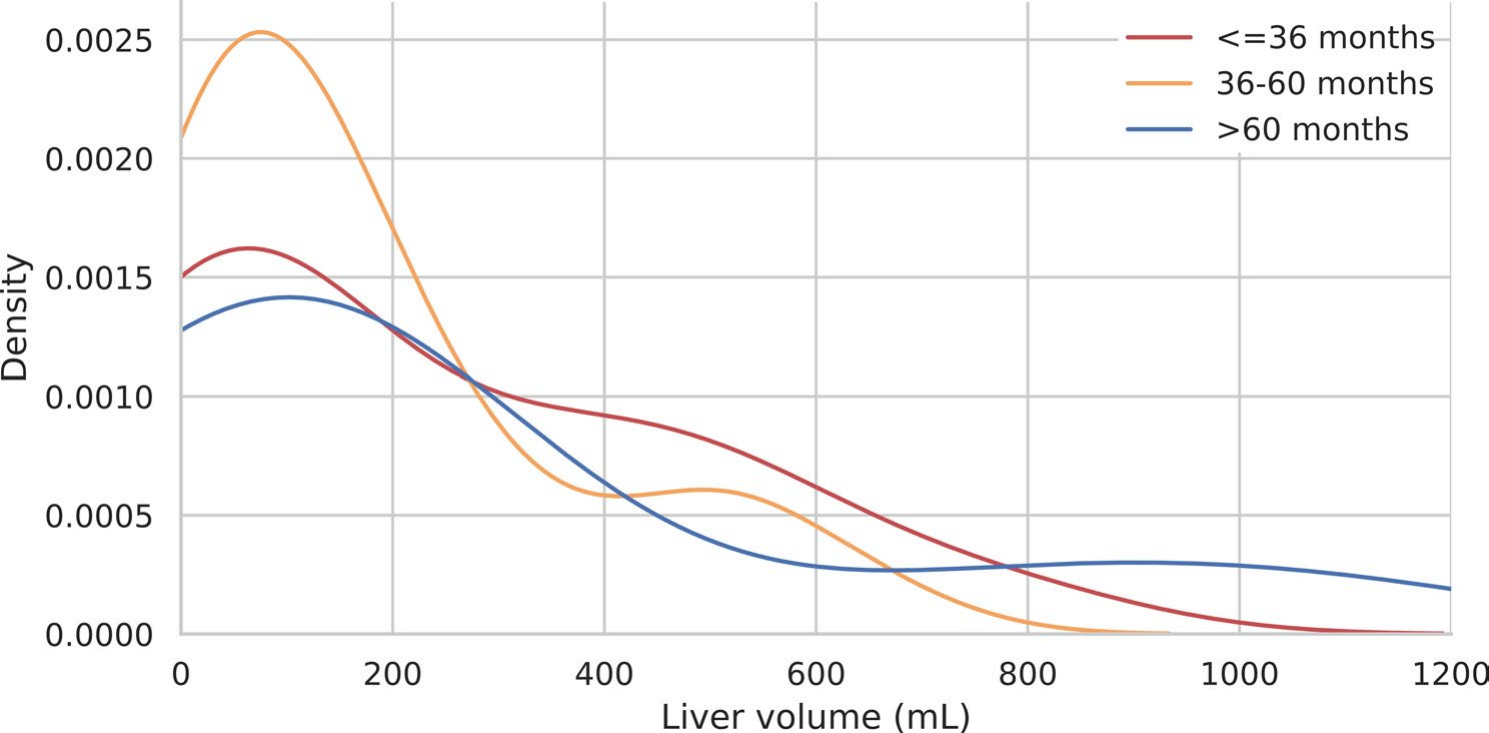
KDE plot for LVs according to the three survival groups above

**Fig. 5:**
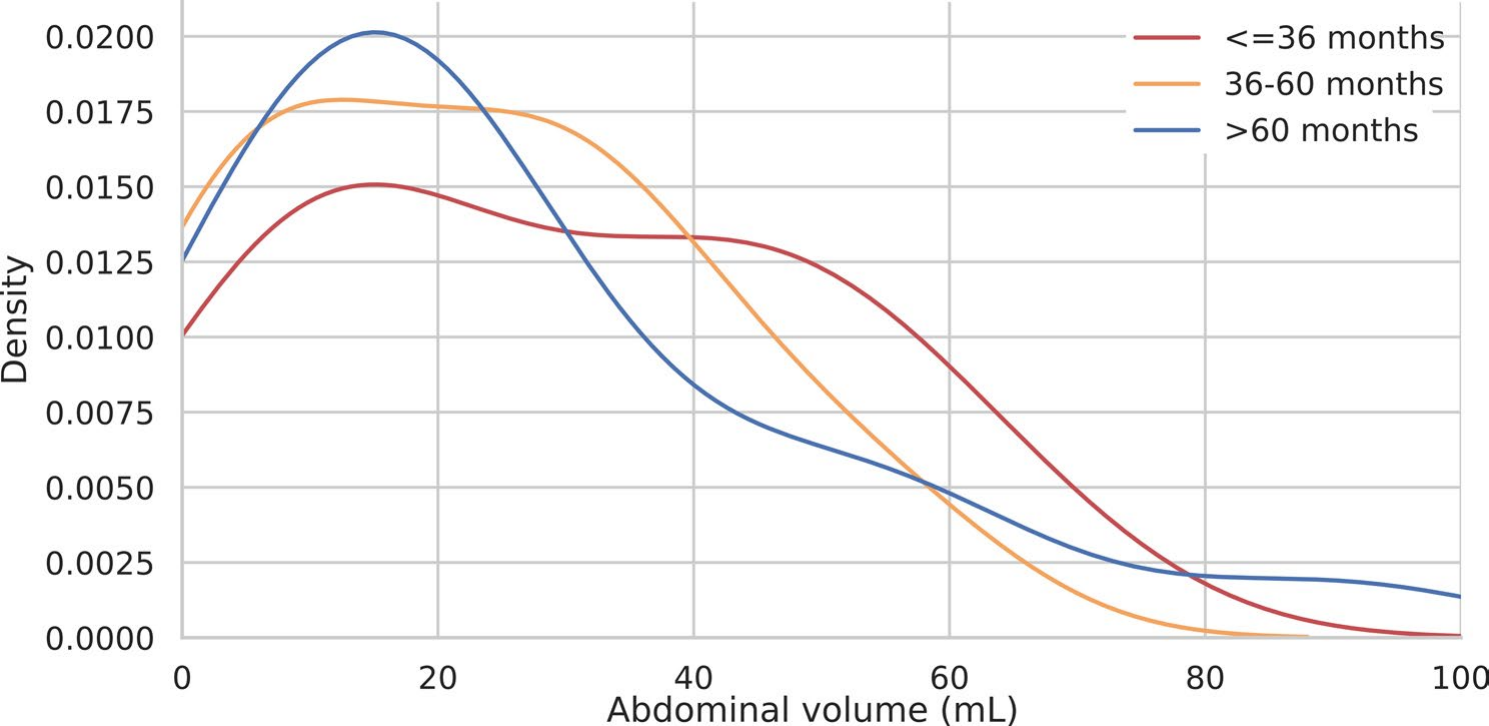
KDE plot for AV according to the three survival groups above

To further explore potential patterns, KDE analysis was applied to liver and abdominal volumes, the most clinically relevant compartments. LV distributions via KDE plots revealed similar patterns across different survival groups with slight variation in density peaks between those surviving less than 36 months and more than 60 months (Fig. 4). AV distributions revealed similar patterns across different survival groups with patients who survived more than 60 months presented a sharp peak below 20 mL, and those who survived 36–60 months presented a wide peak at approximately 10–30 ml. Patients with survival below 36 months displayed more volumes above 30 ml (Fig. 5).

### SUV

#### Survival analysis

For the SUV parameters, we repeated the same analysis process as that used for the volumetric parameters (Cox regression analysis, KM, and KDE analysis) (Table 5).

Univariate Cox regression analysis was performed to evaluated the association between total SUVmean, SUVmeanLiver, SUVmeanAbdomen, SUVmeanBones, and SUVmeanLymphNodes with OS. None of these SUV parameters showed a significant association (*p* > 0.05).

**Table 5:** Cox regression analysis for the SUV parameters

		Cox regression	
Parameter	Mean	*p* value	HR
SUVmean	14.83	0.357	0.978 (0.933–1.025)
SUVmeanLiver	13.82	0.174	0.964 (0.915–1.016)
SUVmeanAbdomen	14.93	0.917	0.998 (0.960.1.037)
SUVmeanBones	11.46	0.201	0.952 (0.882–1.027)
SUVmeanLymphNodes	11.77	0.908	1.005 (0.929–1.087)
SUVmax	40.119	0.128	0.973 (0.94–1.008)
SUVmaxLiver	32.613	0.997	1.000 (9.971–1.029)
SUVmaxAbdomen	30.189	0.533	0.944 (0.974–1.014)
SUVmaxBones	27.563	0.596	0.983 (0.922–1.048)
SUVmaxLymphNodes	24.065	0.973	0.999 (0.96–1.040)

**Fig. 6:**
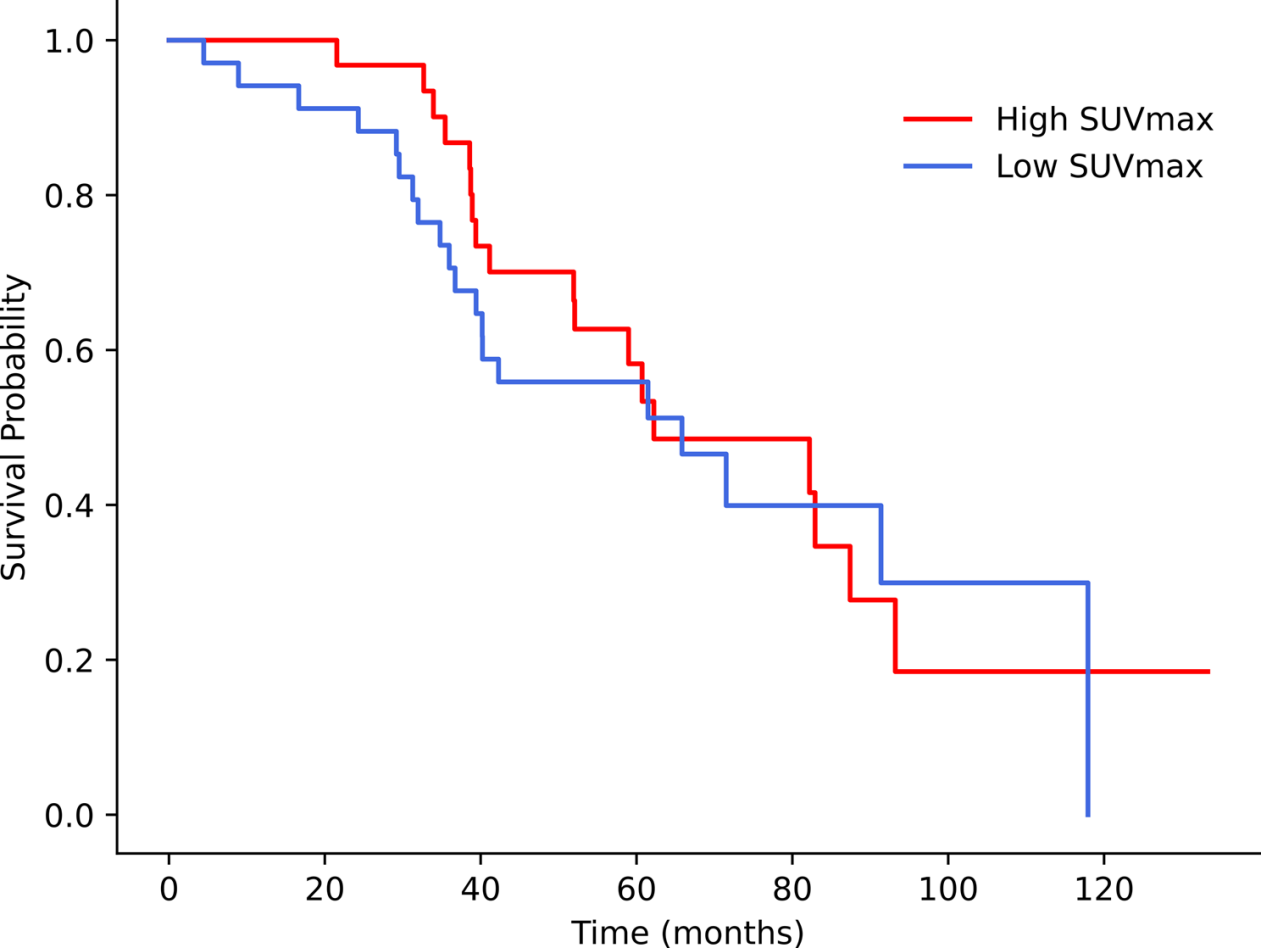
Kaplan‒Meier survival analysis showing the high-SUVmax and low-SUVmax curves

Kaplan‒Meier analysis revealed no statistically significant differences in survival between the groups for any of the SUV parameters analyzed (Table 6; Fig. 6).

**Table 6:** Survival analysis for the SUV parameters

Parameter	Median	Mean Survival(95% CI)low group vs. high group	Survival Rate low group vs. high group	Log-rank – Mantel Cox (*p* value)
SUVmax	36.43	68.22 (53.49–82.95)mo **vs. 73.86 (59.29–88.44)** mo	41.2% **vs. 45.5%**	0.623
SUVmaxLiver	27.12	71.59 (56.05–87.12) mo **vs. 59.23 (49.75–68.72)** mo	45.2% **vs. 35.5**%	0.291
SUVmaxAbdomen	26.63	60.72 (48.32–73.11) mo **vs. 73.53 (57.12–89.95)** mo	46.2% **vs. 40.7%**	0.771
SUVmaxBone	26.51	66.94 (48.66–85.23) mo **vs. 89.42 (66.30-112.53)** mo	33.3% **vs. 57.1%**	0.118
SUVmaxLymphnodes	23.99	68.554 (48.04–89.06) mo **vs. 68.66 (54.92–82.36)** mo	45.8% **vs. 37.5%**	0.918
SUVmean	12.59	70.43 (54.96–85.89)mo **vs. 66.82 (57.04–76.60)** mo	43.2% **vs. 43.3%**	0.769
SUVmeanLiver	11.57	61.05 (50.98–71.11)mo **vs. 67.75 (53.53–81.97)** mo	45.5% **vs. 34.5%**	0.898
SUVmeanAbdomen	13	69.6 (50.06–54.13)mo **vs. 65.29 (54.14–76.43)** mo	46.4% **vs. 40%**	0.851
SUVmeanBone	9.52	73.08 (53.32–92.84)mo **vs. 80.92 (59.08-102.76)** mo	37.5% **vs. 50%**	0.57
SUVmeanLymphnodes	9.39	68.81 (46.65–90.98)mo **vs. 67.88 (55.26–80.49)** mo	37.5% vs. **40%**	0.964

#### KDE analysis for SUV features

**Fig. 7:**
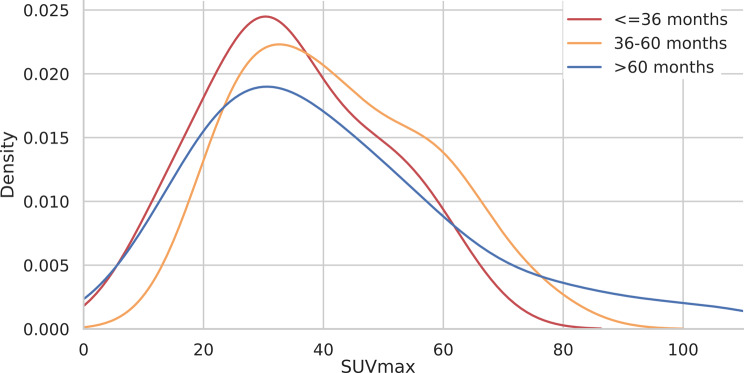
KDE plot for the SUVmax according to the three survival groups above

**Fig. 8:**
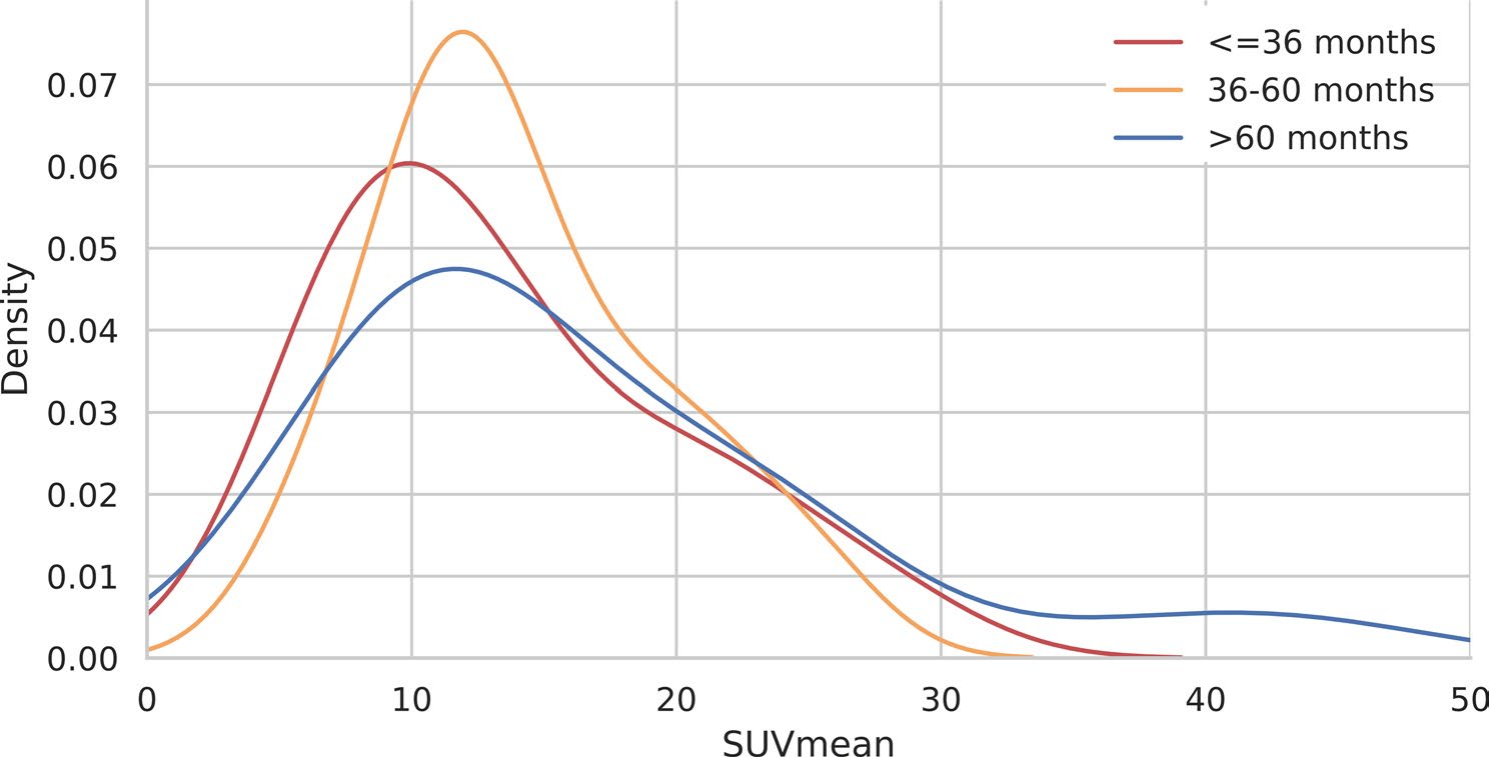
KDE plot for the SUVmean according to the three survival groups above

The analysis of SUVmax distributions via KDE plots revealed similar patterns across different survival groups. All the survival groups presented peak densities of approximately 30. The SUVmean also showed no difference in distribution according to the survival groups, as shown in Figs. 7 and 8.

### Radiomics

#### ROC curve analysis for radiomics features

With respect to the radiomic features, we used receiver operating characteristic (ROC) curves to identify the parameters with the highest prognostic value (survival greater than 36 months and survival greater than 60 months). The top 10 features with the corresponding AUC values are presented in the tables below (Tables 7 and 8).

**Table 7:** Radiomic features with the highest AUC values (event as the survival time > 36 months)

Radiomics Feature	AUC (< 36 Months)
SmallAreaLowGrayLevelEmphasis	0.66
SmallDependenceEmphasis	0.64
SmallDependenceLowGrayLevelEmphasis	0.63
Zone%	0.61
LargeAreaHighGrayLevelEmphasis	0.60
SmallAreaEmphasis	0.60
Idn	0.60
LowGrayLevelZoneEmphasis	0.60
ZoneVariance	0.60

**Table 8:** Radiomic features with the highest AUC values (event as the survival time > 60 months)

Radiomics Feature	AUC (> 60 Months)
DependenceNonUniformityNormalized	0.68
DependenceNonUniformity	0.66
DifferenceAverage	0.64
DifferenceEntropy	0.64
RunPercentage	0.64
Contrast	0.63
Contrast.1	0.63
DifferenceVariance	0.62
JointEntropy	0.62

#### Kaplan‒Meier curves for the 2 best parameters

**Fig. 9:**
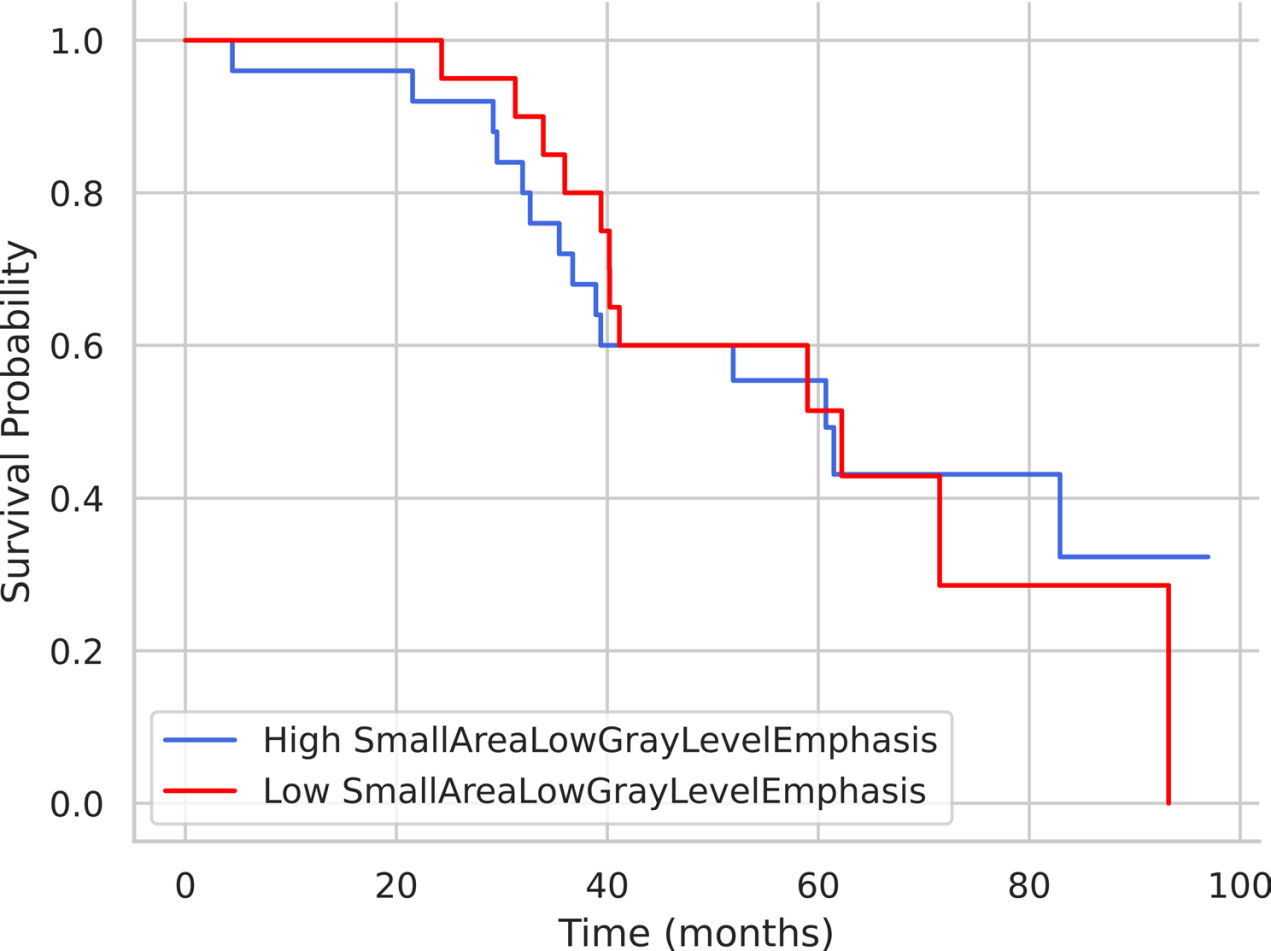
Kaplan‒Meier survival analysis showing high SALGE and low SALGE curves

Given the large number of extracted radiomic features, we decided we selected the most two most promising (based on highest AUC) for Kaplan‒Meier analysis: SmallAreaLowGrayLevelEmphasis (SALGE) and DependenceNonUniformityNormalized (DNUN). Kaplan–Meier analysis revealed no difference in survival between those groups for either parameter (*p* > 0.05) (Fig. 9).

#### KDE analysis for radiomics features

**Fig. 10:**
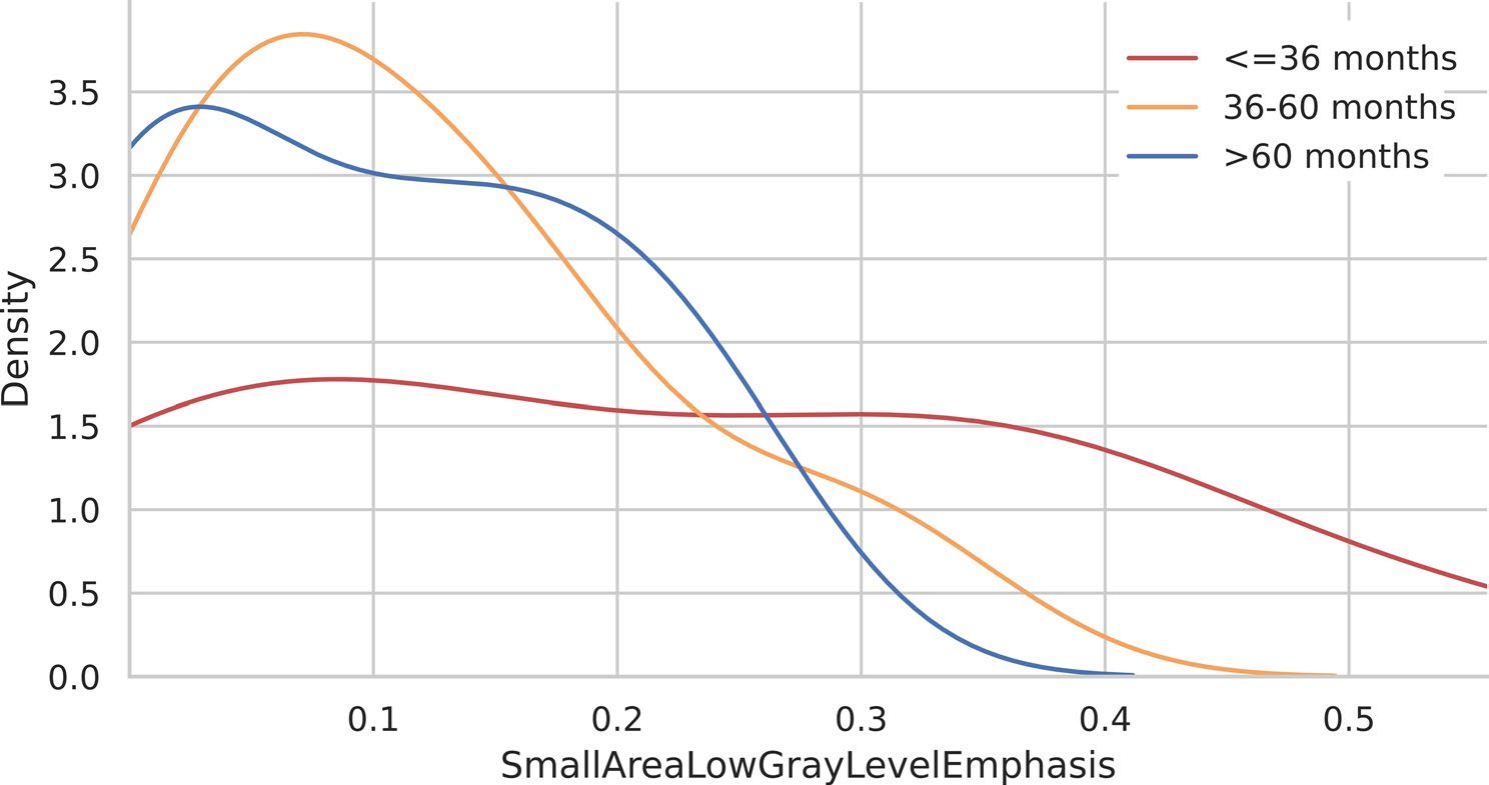
KDE plot for SALGE according to the three survival groups above

**Fig. 11:**
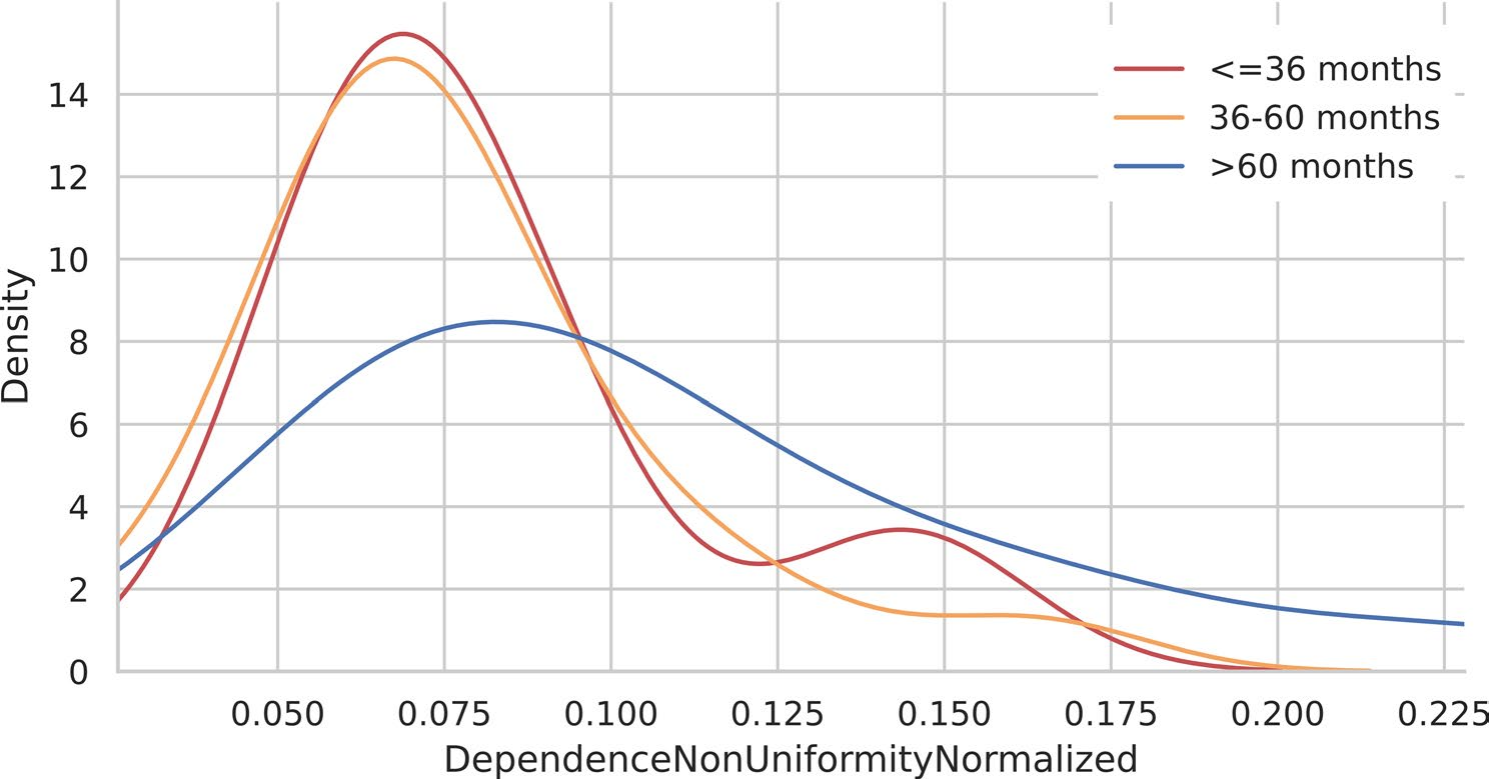
KDE plot for DNUNs according to the three survival groups above

KDE analysis for SALGE revealed a sharp peak density for patients with medium survival and with high survival. Patients with low survival exhibited a flatter and more spread-out distribution without peak densities (Fig. 10). For DNUN, the KDE plots revealed sharp peak densities of approximately for patients with medium and low survival, respectively (Figs 10 and 11).

#### PCA of radiomics features

**Fig. 12:**
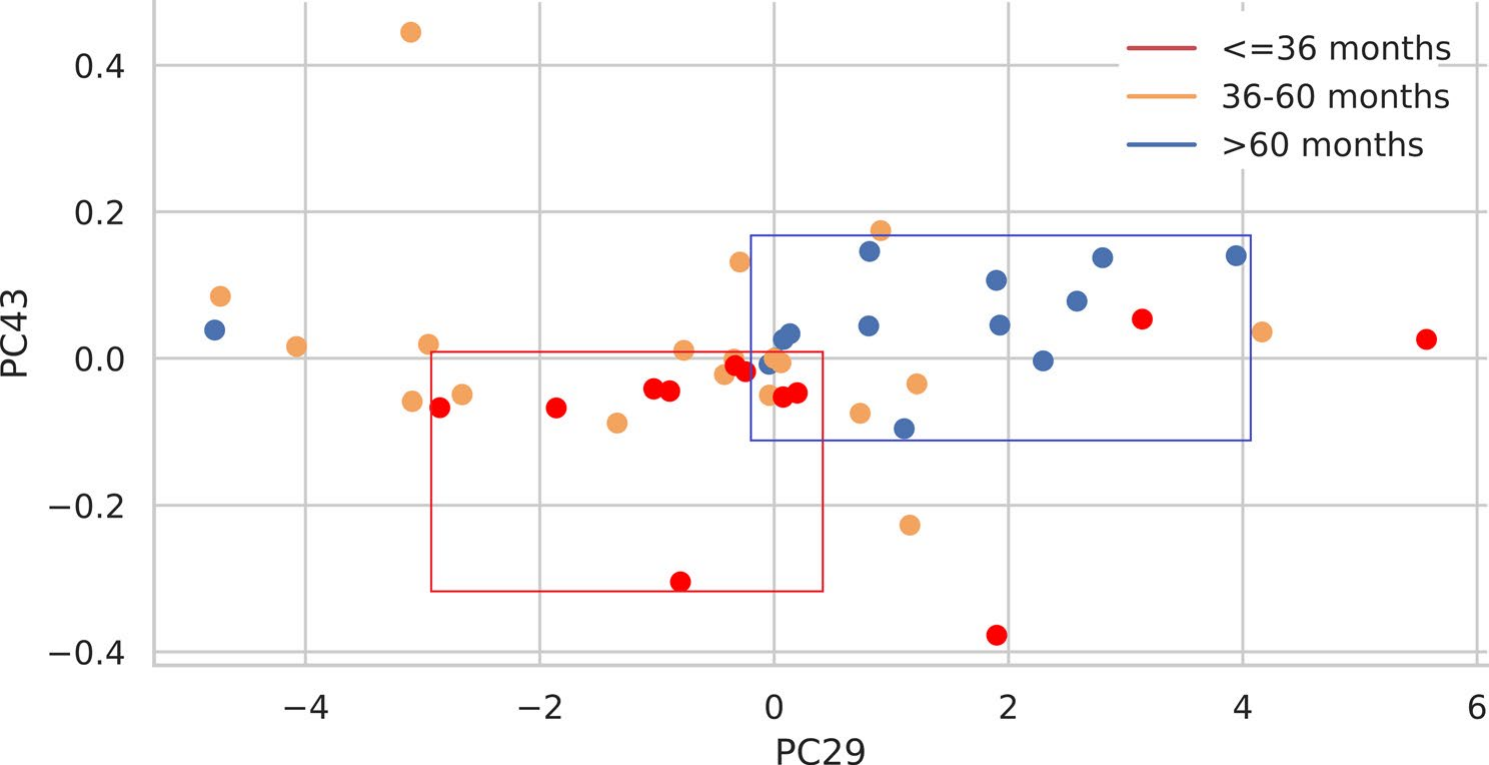
PCA and representation of PC29 and PC43 according to the three survival groups

Finally, we performed principal component analysis (PCA) to reduce the dimensionality of the data and visualize the separation between different survival groups. The scatter plots of principal components (PC29 vs. PC43) revealed distinct groupings on the basis of survival classes, with red, sandy brown, and blue points representing patients with survival classes 0 (low survival), 1 (medium survival), and 2 (high survival), respectively. This analysis revealed a possible grouping for low survival (red rectangle) and another grouping for high survival (blue rectangle). On the other hand, patients with medium survival rates were spread throughout the plot without distinct grouping (Fig. 12).

### Clinicopathological parameters

#### Cox regression

Finally, the clinicopathological parameters yielded no significant results in the Cox regression analysis (Table 9).

**Table 9:** Cox regression analysis of clinicopathological parameters

Parameters	Cox regression analysis	
	p value	HR
Sex	0.244	0.668 (0.338–1.323)
Age	0.803	1.004 (0.975–1.033)
Number of organs affected	0.817	1.042 (0.737–1.47)
Grading	0.966	0.998 (0.435–2.284)
Ki67	0.513	0.974 (0.9-1.054)

Kaplan‒Meier

**Fig. 13:**
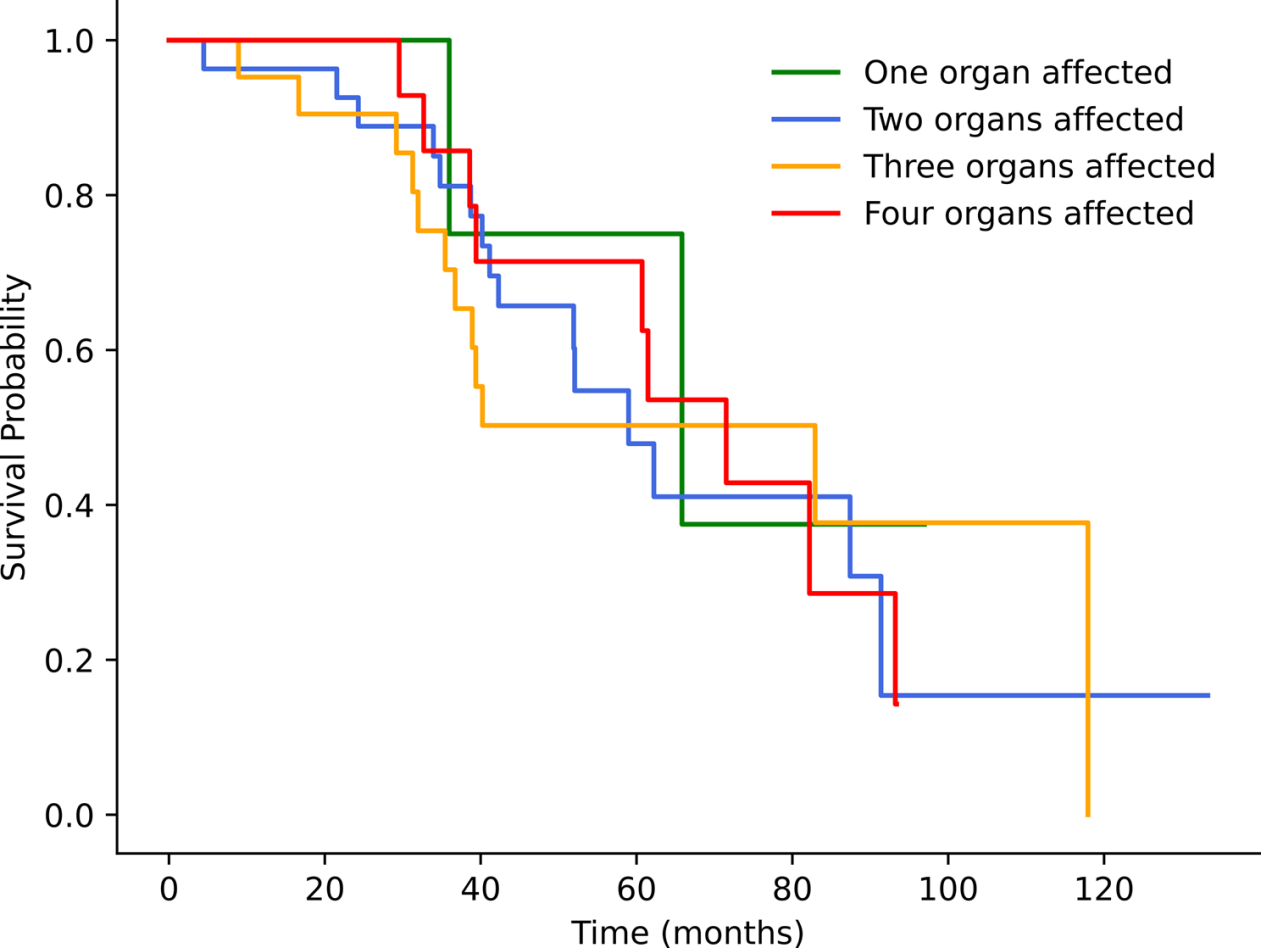
Kaplan‒Meier curves showing the survival differences between patients with different numbers of affected organs

KM analysis revealed no significant difference in survival between the groups in terms of the clinical parameters (Table 10) (Fig. 13).

**Table 10:** Survival analysis for clinicopathological parameters

	Kaplan‒Meier Analysis		
Parameters	**Mean survival (in months)**	**Survival rate**	**Log-rank**
Sex	Females 61.14 (54.76–69.53) **vs. 80.24 (62.21–98.28) Males**	38.5% vs. **50%**	0.244
Number of organs affected	**One organ**: 70.03 (44.87–95.19) **Two organs**: 68.35 (51.02–85.68) **Three organs**: 70.32 (50.62–90.03) **Four organs**: 66.98 (54.35–79.62)	60% 44.4% 42.9% 35.7%	0.924
Grading	Grade 1 63.74 (47.81-79.668) **vs. 69.39 (54-84.79) Grade 2**	50% vs. **35.5%**	0.996
Primary tumor location	**Lungs**: 59.27 (43.81–74.74) **Pancreas**: 67.91 (50.31–85.5) **Small intestine**: 60.58 (40.20-70-95) **Others**: 88.79 (60.18-117.39**)**	50% 33.3% 41.9% 58.3%	0.537

## Discussion

In this study of 67 NET patients undergoing PRRT, we evaluated the prognostic value of PET/CT-derived parameters, including tumor volume, SUV metrics, and radiomic features extracted from liver metastases. Contrary to prior studies, no imaging parameters showed significant associations with OS, nor did clinicopathological factors like age, sex, or number of metastatic organs. These finding underscore the challenge in identifying reliable prognostic markers for treatment response and survival in NET patients treated at specialized centers, where standardized care, relatively uniform baseline characteristics and successful integration of PET/CT imaging into the treatment management may minimize variability in outcomes.

While Pauwels et al. [16] reported that total tumor volume > 670 mL predicted worse prognosis (HR = 3.13, *p* = 0.001), and Ohnona et al. [17] found that pancreatic NET patients with tumor volumes greater than 14 mL had a greater risk of disease progression within two years, our study did not observe such association. This discrepancy may reflect differences in patient selection: our cohort’s mean tumor volume (271 mL) fell below Pauwels’ threshold and above Ohnona’s, potentially limiting prognostic discrimination.

Similarly, our analysis of SUV parameters showed limited prognostic value, with no significant correlation between SUVmax or SUVmean values and OS, aligning with previous finding from Urso et al. [18], which also suggested limited prognostic value of SUV parameters alone.

Our application of radiomics to liver metastases, using volume-weighted averages to represent multiple lesions per patient [19]. Despite modest discriminatory ability in ROC analysis (AUC values of 0.68, 0.66), and some separation of survival groups in KDE analysis, the clinical applicability appears limited. The challenges we encountered may reflect the heterogenous nature of NETs, which presents unique limitations for radiomics analysis compared to other tumor types or are attributed to small cohort size.

Clinicopathological parameters (including sex, age, tumor grade, number of affected organs, and primary tumor location) showed no significant correlation with OS in our cohort. However, our analysis included mostly only grades 1 and 2, limiting the interpretation of Ki67 and grading as prognostic factors.

Given that tracer affinity differs among somatostatin analogs—DOTATOC exhibits more selectivity for SSTR2, while DOTATATE binds to both SSTR2 and SSTR5—our use of ^68^Ga-DOTATOC merits evaluation. Prior research, including Kabasakal et al. (2012), found no significant prognostic differences between DOTATOC and DOTATATE in patient cohorts treated with PRRT, despite the possibility that these changes could affect SUV readings [20].

FDG-avidity is generally linked to tumor dedifferentiation, hence dual imaging using SSTR-PET and ^18^F-FDG-PET may improve prognostic accuracy. According to Chan et al. (2017), discordant imaging—that is, lesions that are both FDG-positive and SSTR-negative—strongly predicts a lower overall survival rate [21]. Even while we only looked at SSTR-PET parameters in our study, further research that uses dual-tracer imaging techniques may improve patient risk assessment and treatment choices.

The retrospective design and small sample size limit our study’s statistical power. Additionally, the indolent and heterogenous nature of NETs and methodological variability in radiomics pose challenges for definitive prognostic assessment. Larger, multicenter studies are needed to validate our findings and support the development of reliable imaging biomarkers in this complex tumor entity.

## Conclusions

In this study, we sought to evaluate the prognostic significance of various DOTATOC PET/CT-derived parameters, including tumor volume, SUV metrics, and radiomic features, in patients with NETs undergoing PRRT. Despite comprehensive analyses, including Cox regression and Kaplan‒Meier survival methods, neither volumetric parameters nor SUV values (SUVmax and SUVmean) demonstrated significant association with OS in this small patient population. Radiomics analysis, while innovative, has also shown limited prognostic value.

These findings suggest that DOTATOC PET/CT scans have limited prognostic value. These findings indicate that large ENETS (European Neuroendocrine Tumor Society) centers already incorporate imaging information, including PRRT cycle frequency, liver-directed therapies, prophylactic surgery, and selective PRRT‒chemotherapy combinations, into personalized treatment plans [21].

## Data Availability

Data are available from the corresponding author on reasonable request.

## Abbreviations

^68^Ga-DOTATOC: Gallium-68-DOTA-Tyr3-octreotide
^177^Lu-DOTATATE: Lutetium-177-DOTA-Tyr3-octreotate
NET: Neuroendocrine tumor
PRRT: Peptide receptor radionuclide therapy
SUV: Standardized uptake value
SUVmax: Maximum standardized uptake value
SUVmean: Mean standardized uptake value
OS: Overall survival
IBI: Inflammation-based index
ALP: Alkaline phosphatase
LDH: Lactate dehydrogenase
KDE: Kernel density estimation
PCA: Principal component analysis
HR: Hazard ratio
CI: Confidence interval
LV: Liver volume
BV: Bone volume
LNV: Lymph node volume
AV: Abdominal volume
LDB: Liver disease burden
ENETS: European Neuroendocrine Tumor Society e.V
